# Quantification of Lateralized Overgrowth and Genotype‐Driven Tissue Composition

**DOI:** 10.1111/cge.14713

**Published:** 2025-02-02

**Authors:** Andrea Gazzin, Giuseppe Reynolds, Damiano Allegro, Davide Rossi, Francesca Sciandra, Hirad Akberi Afkhami, Simona Cardaropoli, Marilidia Piglionica, Nicoletta Resta, Marco Di Stefano, Alessandro Mussa

**Affiliations:** ^1^ Clinical Pediatrics Genetics Unit Regina Margherita Children's Hospital Turin Italy; ^2^ Department of Public Health and Pediatric Sciences University of Torino Turin Italy; ^3^ Postgraduate School of Pediatrics, Department of Public Health and Pediatrics University of Torino Turin Italy; ^4^ Endocrinologia, Diabetologia e Metabolismo U Città della Salute e della Scienza Turin Italy; ^5^ Department of Biomedical Sciences and Human Oncology University “Aldo Moro” Bari Italy

**Keywords:** Beckwith‐Wiedemann spectrum, dual‐energy X‐ray absorptiometry, isolated lateralized overgrowth, lateralized overgrowth, *PIK3CA*‐related overgrowth spectrum, PROS

## Abstract

Lateralized overgrowth (LO) is characterized by excessive growth of one side of the body compared to the other. LO can present as isolated (ILO) or within syndromes, like Beckwith‐Wiedemann Spectrum (BWSp) and *PIK3CA*‐related overgrowth spectrum (PROS). Currently, the diagnosis of LO relies on clinical evaluation and lacks a standardized method. In this study, we evaluated total body dual‐energy X‐ray absorptiometry (TB‐DXA) as a potential tool for standardizing LO assessment. Patients with LO underwent both clinical evaluation and TB‐DXA. TB‐DXA data, including total mass, mass of the three main tissue components (adipose, muscle, and bone), total mass discrepancy ratio, relative tissue composition, and discrepancy of relative tissue composition were calculated and compared with clinical findings. Differences between affected regions and the contralateral side were assessed. A total of 46 patients (61% PROS, 24% BWSp, 15% ILO) were included in this study. TB‐DXA detected overgrowth regions aligned with clinical evaluation in 91% of cases and was able to identify localized overgrowth even when clinically overlooked. Additionally, TB‐DXA revealed differences in tissue composition between affected and unaffected regions for symmetrical body areas, with these differences varying by diagnostic subgroup. Different patterns of tissue composition overgrowth were observed among different conditions, with PROS predominantly showing adipose tissue overgrowth, while BWSp/ILO mainly osteo‐muscular overgrowth. TB‐DXA is an accurate, safe, and reproducible tool in the clinical setting providing an objective method for identifying and quantifying LO. It offers valuable guidance for clinicians in the diagnosis and management of LO.

## Graphical abstract

Lateralized overgrowth involves asymmetric body growth across conditions. TB‐DXA accurately identifies and quantifies tissue overgrowth, showing varied patterns among conditions and improving diagnosis.

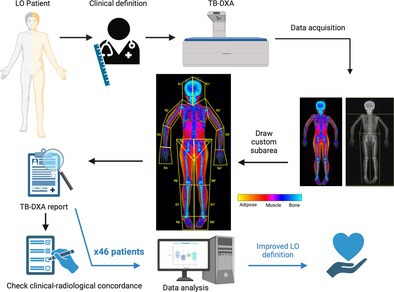

## 1 Introduction

Lateralized overgrowth (LO), also known as segmental overgrowth, is a condition characterized by excessive growth of any region of the body compared to the corresponding contralateral region. LO is defined isolated (ILO) when occurring without other clinical features, developmental disorders, anomalies, or malformations, and without a detectable molecular cause [1, 2, 3]. Conversely, LO can belong to a syndromic spectrum and be part of the phenotype of several syndromic conditions. Among these, Beckwith‐Wiedemann spectrum (BWSp) is the most common, affecting 1 in 10 000 live births [4]: LO is a key feature of BWSp, as it is present in more than 65% of patients [5, 6, 7]. BWSp is caused by epigenetic alterations affecting two imprinting centers (ICs) on chromosome 11p15.5 [8, 9, 10]. *PIK3CA*‐related overgrowth spectrum (PROS) is the second most common cause of syndromic LO, with a prevalence of 1:25 000 live births [11]. PROS encompasses various clinical phenotypes characterized by localized overgrowth resulting from somatic hyperactivating variants in the *PIK3CA* gene, leading to cellular proliferation [12]. The heterogeneous phenotypes of PROS arise from the extent of somatic mosaicism and the activating strength of the variant [13]. Several other genes involved in or interacting with the PI3K/AKT/mTOR pathway can also lead to syndromic LO overlapping with PROS [13], such as *AKT3* [14] and *MTOR* [15].

Slight differences in size between symmetrical parts of the human body are normal, making the assessment and quantification of segmental overgrowth a critical challenge. Although measurable directly or through imaging techniques, LO is usually assessed clinically (i.e., “from the end of the bed”) [16]: a theoretical 10% cut off in the measured/perceived body part size (e.g., girth or length of a limb) is adopted to define overgrowth and frame it as pathologic. However, in clinical practice, quantifying the degree of LO is challenging due to its heterogeneous variability in anatomical location and extent. Imaging techniques can be used to define LO as well as characterize its severity, body extension, and tissue composition [17]. Even more complicated is to monitor evolution over time, especially in growing children. Either X‐ray/computer tomography scan, ultrasound or magnetic resonance imaging can be used, depending on the anatomical region and the type of tissue involved. Each method has limitations: X‐ray cannot evaluate soft tissues and magnetic resonance imaging, while optimal for morphological definition, is costly, time‐consuming, and often requires sedation for younger patients [18, 19]. Moreover, measurements with such techniques mostly are operator‐dependent and pose difficulties in monitoring the variable evolution of LO during childhood growth. With this respect, it should be emphasized how bodily modifications in terms of size and body parts composition can themselves undergo changes because of the child's growth process: these are not fully defined and are often not taken into consideration during clinical assessments of LO.

In this context, total body dual‐energy X‐ray absorptiometry (TB‐DXA) offers many advantages: it is safe, reproducible, fast, non‐operator dependent, and cost‐effective. Most importantly, TB‐DXA, by measuring how different body tissues (adipose, muscle, and bone) absorb X‐rays, allows for the precise quantification of respective proportions [20] and demonstrated to be a reproducible method with little variation within and between observers [21]. It provides measurements at both whole‐body and customizable regional levels [22], which can be compared with the corresponding contralateral body part. Radiation exposure is minimal [23] with no absolute contraindications except for pregnancy.

Parker et al. used dual‐energy X‐ray absorptiometry (DXA) to assess the efficacy of low‐dose sirolimus in individuals with PROS. They compared affected and unaffected body areas in 23 participants across three time points. The study found a significant reduction in the volume of affected tissues during sirolimus treatment, with a trend toward greater volume reduction in participants with predominant adipose overgrowth [24].

In this study we standardized the approach to LO by TB‐DXA to define, quantify, and characterize the heterogeneous clinical presentations including of both ILO and syndromic LO, in children and adults. Our primary objective was to determine whether TB‐DXA can accurately estimate differences in mass between the affected body regions and the corresponding contralateral non‐affected regions, and to assess the consistency of these findings with clinical evaluations. Additionally, we analyzed differences in tissue overgrowth patterns in the affected regions across various diagnoses.

## 2 Methods

### 2.1 Patients

A single‐center study was conducted on patients with overgrowth of at least one body segment, either isolated or syndromic. The study was approved by the local Ethics Committee (Protocol No. 35286) and patients were enrolled after obtaining informed consent from the patient or their parents/guardian. For each patient, demographic data, age, sex, clinical characteristics, clinical diagnosis, molecular analyses performed, information on the LO region, and the final diagnosis were collected. BWSp diagnosis was assessed according to clinical criteria [25] and molecularly confirmed by methylation tests at IC1 and IC2 chr11p15.5 on DNA extracted from blood. Cases with paternal uniparental disomy of chr11p15.5 were confirmed by microsatellite/single nucleotide polymorphisms analysis. If negative, the test was repeated on DNA extracted from affected tissue. PROS patients were diagnosed according to clinical criteria [26] and molecularly confirmed with a high‐depth (> 1000 reads) next generation sequencing (NGS) customized panel of genes including *PIK3CA*, on DNA extracted from affected tissue, as described elsewhere [13]. ILO was defined as patients without any additional or syndromic clinical characteristics besides LO and tested with all of the above molecular analysis with negative test results.

### 2.2 Clinical Definition of LO

For each overgrowth region, a measurement was obtained when feasible, to assess asymmetry with respect to the contralateral body region. Clinically, a 10% cut off in measurement was defined as significant (e.g., girth and/or length when referring to limbs). When not measurable (e.g., in some cases with overgrowth of the head/face or pelvic region), asymmetry was considered as significant based upon the discretion of the examining clinician [16].

### 2.3 TB‐DXA

**FIGURE 1:**
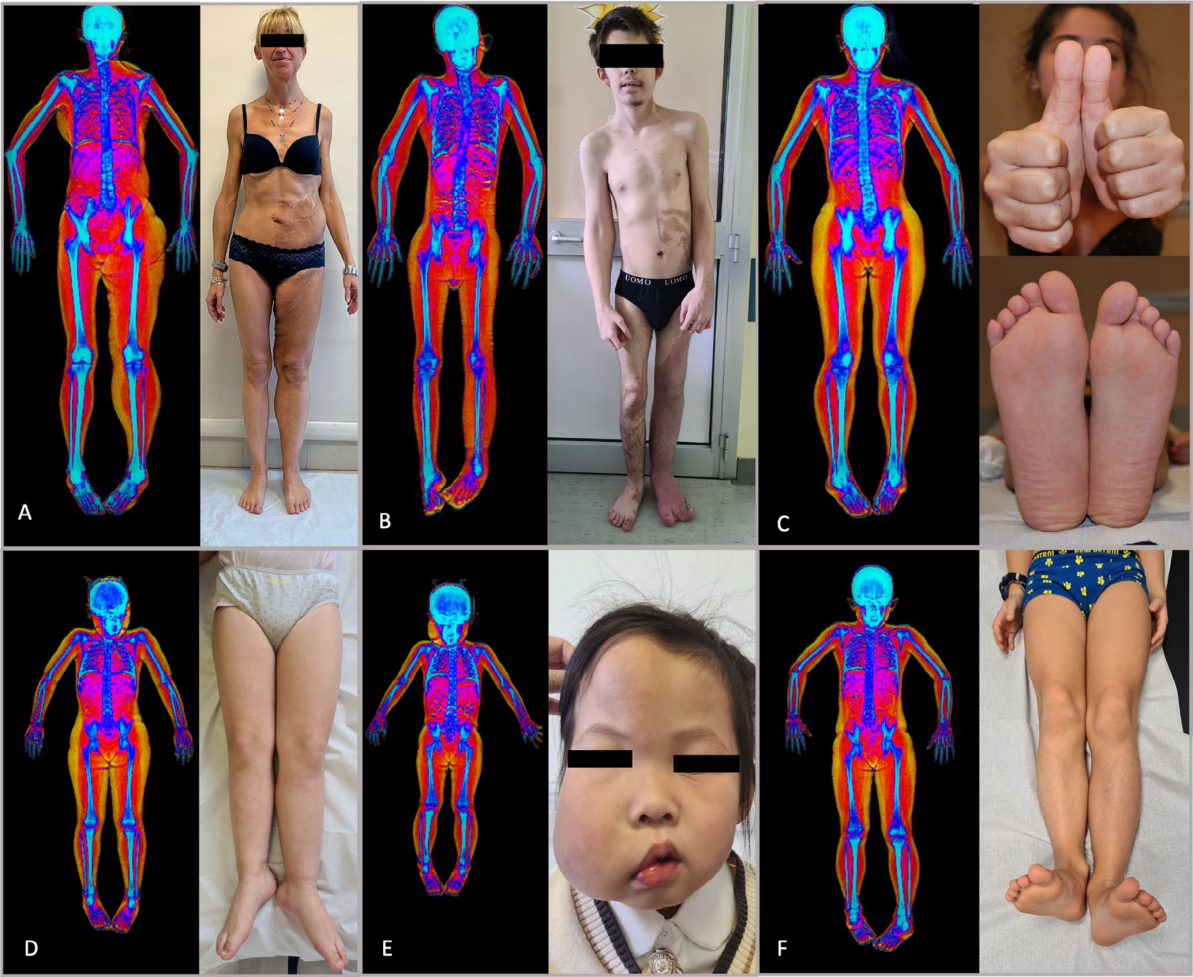
Total body dual‐energy X‐ray absorptiometry images and corresponding clinical photography of the LO affected region(s), together with the corresponding contralateral unaffected region(s) of patients affected by lateralized overgrowth (LO) of different body regions. (A) Patient affected by *PIK3CA*‐related overgrowth spectrum (PROS) with lateralized overgrowth and lipomatous infiltration of left lower limb, hemiabdomen, hemihead, and thorax; (B) Patient affected by PROS with LO of the left hemihead, right upper limb and left lower limb with left splayed foot, extensive vascular malformation of left lower limb and left hemiabdomen; (C) Patient affected by Beckwith‐Wiedemann spectrum (BWSp) with LO involving the entire right hemibody; (D) Patient affected by BWSp with LO involving the left lower limb; (E) Patient affected by PROS with right hemihead LO due to facial infiltrating lipomatosis; (F) patient affected by isolated LO involving left lower limb.

All participants underwent TB‐DXA by Horizon DXA System, Hologic 2520 (Santa Clara, CA 95054). To analyze precisely the body regions affected by LO, tailored data was collected for each patient by breaking out DXA readings in specific subregions based on the respective overgrowth body region defined clinically. The total‐body scan area was divided into 16 regions: R1‐8 of one side, and their corresponding contralateral (R1′‐8′). The regions considered were hemihead, hemipelvis (from the base of the femoral neck to the upper edge of the iliac crest), thigh (from the tibial plateau to the base of the femoral neck), leg (from the tibia‐calcaneal articulation to the tibial plateau), foot, arm (from the elbow joint to the humeral‐scapular joint), forearm (from the wrist joint to the elbow joint), hand. Hemithorax and hemiabdomen were not considered as the laterality of the organs contained generates by itself a discrepancy in mass and tissue compositions among the two sides. Based on clinical assessment, each of the 16 regions were classified as “affected” or “not affected” by overgrowth (Table S1, Figure 1).

### 2.4 Data Analysis

To determine the amount of tissue affected by LO, the total mass (*m*_t_) in grams detected by TB‐DXA in each region was recorded: *m*_t_ corresponds to the sum of the mass of the three main components (adipose—*m*_A_‐, muscle—*m*_M_‐, and bone—*m*_B_‐tissue) of each of the body region(s) defined. Each of the 16 body regions was defined as “affected” or “unaffected” based on clinical evaluation. To assess if TB‐DXA allows the identification of body regions with overgrowth and whether physiological differences in body laterality were relevant, we compared both overgrowth and unaffected regions of the same side of the body with the contralateral unaffected specular body regions. To assess the degree of concordance between TB‐DXA readings and clinical detection of LO, the discrepancy ratio in *m*_*t*_ between the affected and the corresponding contralateral unaffected region(s) was calculated and a *m*_t_ discrepancy cut off of 10% was set to define as significant a difference.

$$m_{t}\text{discrepancy ratio}\;\left(\%\right)=\frac{m_{t}\;\text{affected}\;\left(g\right)-m_{t}\;\text{contralateral homologous}\;\left(g\right)}{m_{t}\;\text{contralateral homologous}\;\left(g\right).}$$

Subsequently, the relative composition (RC) of each tissue was calculated as the mass of the tissue of interest (adipose—*m*_A_‐, muscle—*m*_M_‐, bone—*m*_B_‐tissue) individually taken and divided by the *m*_t_. RC was calculated for each region (R1‐8 and R′1–8) and for each of the tissue: bone (RC_B_), muscle (RC_M_) and adipose tissue (RC_A_).

$${\mathrm{RC}}_{A}=\frac{\sum m_{A}}{\sum m_{t}}\;\;\;\;\;{\mathrm{RC}}_{M}=\frac{\sum m_{M}}{\sum m_{t}}\;\;\;\;\;{\mathrm{RC}}_{B}=\frac{\sum m_{B}}{\sum m_{t}}$$

To define which tissue was mainly affected by the overgrowth, the RC discrepancy (ΔRC) was calculated. ΔRC is the difference of RC of the region(s) affected by LO (RC) and the corresponding contralateral unaffected region(s) (RC′). ΔRC was calculated for each tissue: bone (ΔRC_B_), muscle (ΔRC_M_), and adipose tissues (ΔRC_A_).

$${\mathrm{\Delta RC}}_{A}={\mathrm{RC}}_{A}-{\mathrm{RC}}_{A}^{\prime }\;\;\;\;\;{\mathrm{\Delta RC}}_{M}={\mathrm{RC}}_{M}-{\mathrm{RC}}_{M}^{\prime }\;\;\;\;\;{\mathrm{\Delta RC}}_{B}={\mathrm{RC}}_{B}-{\mathrm{RC}}_{B}^{\prime }$$

**FIGURE 2:**
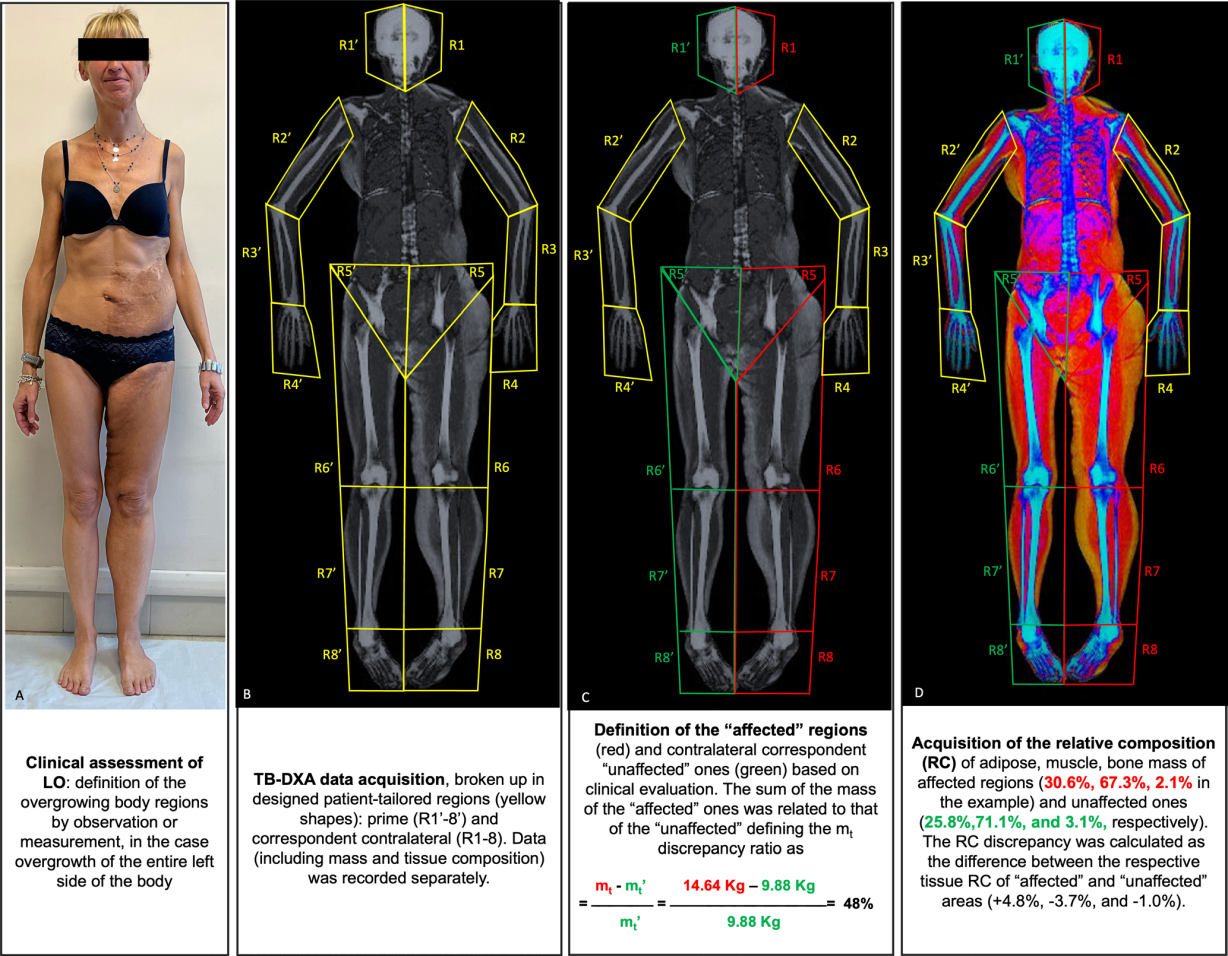
Workflow of total body dual‐energy X‐ray absorptiometry study of LO of patient #17. (A) Clinical assessment of LO. (B) TB‐DXA image acquisition and patient‐tailored regions design (yellow shapes). Prime (R1′‐8′) regions represent the corresponding contralateral of R1‐8. (C) Based on clinical evaluation, each of the 16 body regions were defined as “affected” (red shapes) or “contralateral unaffected” (green shapes). For each “affected” region, the total mass (*m*_t_) was recorded and compared with the correspondent contralateral regions (*m*_t_′) to assess the *m*_t_ discrepancy ratio. (D) For each of the 16 body regions, the mass of fat, lean, and bone tissue was also recorded and the relative composition (RC) of each was calculated. Each RC of the affected body region was then compared with the correspondent contralateral unaffected region.

For patients presenting with LO affecting multiple body regions, the absolute mass, the RC and ΔRC were calculated by summing values of each region, both for the affected (R1 to 8) and unaffected sides (R′1 to 8). The higher ΔRC of the affected region was considered as a marker of the predominant overgrown tissue (Figure 2).

### 2.5 Statistical Analysis

Homoscedasticity of data was first assessed by the Shapiro–Wilk test. Fisher's exact test was used for categorical data to assess associations between variables. The unpaired Student's *t*‐test was applied to compare the means of two groups, while the paired *t*‐test was used for analyzing differences between matched pairs. Differences in means across three or more groups were tested by one‐way ANOVA. The Fisher's exact test was performed to determine differences in the prevalence of a specific tissue ΔRC across the various diagnostic subgroups (BWSp, ILO, and PROS). ΔRCs values for each tissue were the compared among the diagnoses subgroups also with unpaired Student's *t*‐test. Statistical significance was defined as a *p* value of less than 0.05 for all tests. Statistical analyses were performed with GraphPad Prism version 10.0.0 for Windows, GraphPad Software, Boston, Massachusetts USA, www.graphpad.com. Figures were created with BioRender.com.

## 3 Results

We evaluated 46 patients, 21 males, and 25 females, with a mean age of 12.9 ± 9.9 years (median 11.0 years, interquartile range [IQR] 9 years). About 28 patients (61%) were diagnosed with PROS, 27 of them molecularly confirmed and 1 clinical diagnosis with multiple negative molecular tests. PROS patients were 14 males and 14 females, with a mean age 15.4 ± 11.6 years (median age of 12.0 years, IQR 14.3 years). 11 patients (24%) were diagnosed with BWSp, 7 molecularly confirmed, and 4 clinically diagnosed according to the diagnostic criteria [25]. In the BWSp subgroup, three subjects were males and eight were females, with a mean age of 8.8 ± 4.2 years (median age 8.5 years, IQR 6.5 years). Finally, 7 patients (15%) were diagnosed with ILO, four of which were males and three females, with a mean age of 8.9 ± 3.0 years (median age of 9.0 years, IQR 5.0 years).

### 3.1 TB‐DXA Detects Overgrowth Region(s), is Concordant With Clinical Evaluation and More Precise Than Clinical Assessment

Differences in the *m*_t_ of the affected region(s) compared with the corresponding contralateral one(s) were all statistically significant for all the disease subgroups (namely *p* < 0.001 in PROS, *p* = 0.021 in BWSp, and *p* = 0.033 in ILO, paired *t*‐student). Contrarily, the comparison of *m*_t_ between the two corresponding contralateral sides of the unaffected region(s) of the body retrieved no significant differences, confirming the clinical finding of absent LO of these regions.

In 91% of cases, the region(s) identified as affected by overgrowth during clinical evaluation was also confirmed by TB‐DXA, indicating a high level of agreement between clinical evaluation of LO and TB‐DXA. Furthermore, in the remaining 9% of cases, TB‐DXA identified a difference in *m*_t_ discrepancy ratio compared to the contralateral limb, although it did not meet the 10% threshold for the definition of LO: for 3 of these patients, *m*_t_ discrepancy ratio was slightly below the threshold (8%, 7%, and 9% in patients 16, 33, and 38, respectively).

Moreover, TB‐DXA identified localized overgrowth even when clinically overlooked: in two cases (4.3%) it revealed a significant *m*_t_ discrepancy ratio of region(s) not detected as affected at clinical examination. In the first case (patient 46), LO was referred at clinical evaluation as confined to the right side of face and head: TB‐DXA confirmed this finding, but also revealed a significant LO of right upper limb with corresponding *m*_t_ discrepancy ratio of 18%. Clinical re‐evaluation of the patient confirmed a slight girth discrepancy of the right arm and forearm. In the other case (patient 37), TB‐DXA confirmed the clinically defined LO regions (right lower limb) also revealing left upper limb LO with corresponding *m*_*t*_ discrepancy ratio of 11%, subsequently confirmed at clinical re‐evaluation.

### 3.2 Affected Region(s) and Unaffected Contralateral Homologues Ones(s) for Symmetrical Body Structures Have Different Tissue Composition

**FIGURE 3:**
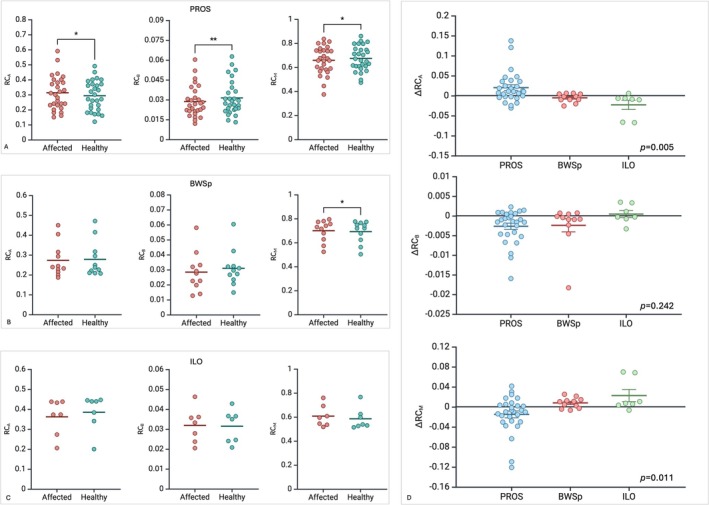
Dot plots showing comparisons between relative composition (RC) of the LO affected region(s) and the contralateral healthy corresponding contralateral in (A) PROS patients, (B) BWSp patients, and (C) ILO patients. *p < 0.05, **p < 0.01 by paired Student *t*‐test. (D) Dot plots showing comparison of difference in RC (ΔRC) between diagnostic groups (one‐way ANOVA). RC_A_: Relative Composition of the adipose tissue; RC_B_: Relative composition, bone tissue; RC_M_: Relative composition, muscle tissue; ΔRC_A_: Relative composition discrepancy, adipose tissue; ΔRC_B_: Relative composition discrepancy, bone tissue; ΔRC_M_: Relative composition discrepancy, muscle tissue.

TB‐DXA identified significant differences in tissue composition between the affect region(s) and the healthy corresponding contralateral ones, in terms of RC. In particular, in PROS patients, differences in RC were significant for all the three tissues measured by TB‐DXA, (RC_Bone_ *p* = 0.009, RC_Adipose_ *p* = 0.010, and RC_Muscle_ *p* = 0.024, respectively, by paired *t*‐student). In BWSp patients, TB‐DXA found statistically significant differences in RC_M_ (*p* = 0.036), but not in RC_B_ and RC_A_. Finally, no statistically significant differences in RC were identified in the ILO subgroup. Tissue composition across the three diseases is summarized in Figure 3A–C and Table 1. By aggregating data of BWSp and ILO patients, we found statistically significant differences in RC_M_ (*p* = 0.020) and RC_A_ (*p* = 0.036) but not in RC_B_.

**TABLE 1:** Summary of relative composition and Δ relative composition mean values among diagnostic subgroup.

Patient group	*n*	RC LO affected region(s) mean ± SD	RC contralateral unaffected corresponding contralateral region(s) mean ± SD	*p*	ΔRC mean ± SD
PROS	28	Adipose: 31.3% ± 11.2%	Adipose: 29.2% ± 10.0%	*p* = 0.010	Adipose: 2.0% ± 3.8%
		Muscle: 65.8% ± 11.4%	Muscle: 67.4% ± 10.2%	*p* = 0.024	Muscle: −1.5% ± 3.6%
		Bone: 2.8% ± 1.2%	Bone: 3.1% ± 1.3%	*p* = 0.009	Bone: −0.3% ± 0.4%
BWSp	11	Adipose: 27.3% ± 8.50%	Adipose: 27.75% ± 8.98%	—	Adipose: −0.5% ± 1.1%
		Muscle: 69.9% ± 8.9%	Muscle: 69.2% ± 9.1%	*p* = 0.036	Muscle: 0.8% ± 1.0%
		Bone: 2.8% ± 1.3%	Bone: 3.1% ± 1.2%	—	Bone: −0.2% ± 0.6%
ILO	7	Adipose: 36.1% ± 9.1%	Adipose: 38.4% ± 9.1%	—	Adipose: −2.3% ± 3.0%
		Muscle: 60.7% ± 8.9%	Muscle: 58.4% ± 9.0%	—	Muscle: 2.3% ± 3.2%
		Bone: 3.2% ± 0.9%	Bone: 3.1% ± 0.8%	—	Bone: 0.1% ± 0.2%
Total	46	Adipose: 31.0% ± 10.5%	Adipose: 30.2% ± 10.1%	—	Adipose: 0.7% ± 3.6%
		Muscle: 66.0% ± 10.7%	Muscle: 66.4% ± 10.2%	—	Muscle: −0.4% ± 3.4%
		Bone: 2.9% ± 1.1%	Bone: 3.1% ± 1.2%	—	Bone: −0.2% ± 0.4%

Abbreviations: BWSp: Beckwith‐Wiedemann spectrum, ILO: Isolated lateralized overgrowth, PROS: *PIK3CA*‐related overgrowth spectrum, RC: Relative composition, SD: Standard deviation, ΔRC: Relative composition discrepancy.

### 3.3 TB‐DXA Reveals Different Patterns of Composition of Tissue Overgrowth of the Affected Region(s) in PROS, BWSp, and ILO

ΔRC_A_ was significantly different among PROS, BWSp, and ILO (*p* = 0.005). In details, ΔRC_A_ was higher in the PROS subgroup compared to both patients with BWSp (*p* = 0.037) or ILO (*p* = 0.009). On the other hand, no difference was found between the BWSp and ILO subgroups. Also, ΔRC_M_ was significantly different among the three diagnostic subgroups (*p* = 0.011): ΔRC_M_ was significantly lower in the PROS subgroup compared to both patients with BWSp (*p* = 0.0470) and ILO (*p* = 0.0172), while no significant difference was found comparing the BWSp and ILO subgroups. The comparison of ΔRC_B_ tissue did not reveal any statistically significant differences in the PROS, BWSp, and ILO groups (Figure 3D).

## 4 Discussion

As the quantification and definition of LO currently rely primarily on clinical evaluation, and its characterization is hampered by the lack of a standardized method, this study aimed to standardize the approach for assessing and characterizing LO, despite the challenges posed by the extreme heterogeneity of cases. To address the difficulties encountered in clinical practice—such as the absence of standardized methods for repeated measurements, lack of reference values for different body parts, heterogeneity of overgrown areas in LO patients, and the ever‐changing body size in growing children—we chose to compare symmetrical body parts in a self‐controlled manner. Our data confirmed that TB‐DXA is a very reliable and precise tool to assess mass discrepancy and differences in tissue composition in such cases, allowing a precise estimation of differences between LO‐affected regions and the contralateral unaffected corresponding contralateral ones. TB‐DXA identification of differences was consistent with clinical evaluation and proved more precise and sensitive in detecting small differences in body parts. Indeed, in some cases TB‐DXA identified overgrown regions that were initially overlooked and underestimated at the first clinical evaluation, which were subsequently confirmed clinically. Most importantly, this study is the first to demonstrate that the three most common forms of LO (PROS, BWSp, and ILO) exhibit significant differences in tissue composition within the affected body area. Regardless of the specific body areas involved, LO regions in patients with PROS typically show a predominance of adipose tissue over muscle and bone, confirming preclinical data [27]. BWSp and ILO show a very similar tissue composition of the LO areas, predominantly featuring bone and muscle overgrowth. This finding has relevant clinical implications as it may reflect the different disabilities and symptoms observed in patients with PROS and BWSp.

While TB‐DXA demonstrated high performance in defining LO, it has several limitations. One is its low spatial resolution: although it provides whole‐body or regional composition assessments, it does not allow for detailed tissue distribution analysis. Parallelly, it has poor ability in soft tissue discrimination: although it excels at differentiating between bone, lean mass, and fat mass, it has limited capability to further distinguish between different types of lean tissue, such as differentiating muscle from organ tissue. Finally, although the radiation dose from DXA is very low (about 0.00893 milliSieverts) [23], it still involves exposure.

This study has few limitations to consider. First, TB‐DXA was evaluated across a diverse patient group ranging from pediatric to young adult ages: it is possible that results can differ based on sex, age, pubertal development, and other factors not considered in this study. Nevertheless, despite the differences in body composition across different life stages and between sexes [28], our analyses compared regions within the same individual, thus eliminating interindividual variability such those. Furthermore, physiological discrepancy between symmetrical regions of the body is possible and considered non‐pathologic. Yet, by making a comparison between corresponding contralateral unaffected regions, we showed that these differences were not due to random physiological discrepancies between the two hemisides of the body. The sample size was small, a factor that might have invalidated the statistical power of some comparisons: increasing the sample size in future studies is desirable.

## 5 Conclusion

In conclusion, TB‐DXA is safe, quick, and requires minimal patient involvement or preparation, with limited radiation exposure. TB‐DXA allows a standardized approach to the definition and characterization of LO in the clinical setting, providing the bases for monitoring patients over time and identifying differences in tissue composition in overgrowth areas. This approach has allowed us to identify distinct patterns of tissue overgrowth in different diseases: predominantly adipose in PROS, and primarily muscle‐skeletal in BWSp and ILO. Future studies will further explore these differences and their potential clinical implications, as well as define optimal strategies for precisely monitoring the evolution of LO over time and the outcomes of treatment.

## Author Contributions

A.G., M.D.S., and A.M. conceptualized and designed the study, drafted the initial manuscript, and critically reviewed and revised the manuscript. G.R. drafted the initial manuscript, and critically reviewed and revised the manuscript. D.A., D.R., F.S., and H.A.A. designed the data collection instruments, collected data, carried out the initial analyses and critically reviewed and revised the manuscript. S.C., M.P., and N.R. critically reviewed and revised the manuscript. All authors approved the final manuscript as submitted and agree to be accountable for all aspects of the work.

## Ethics Statement

The study was approved by the Institutional Review Board of A.O.U. *Città della Salute e della Scienza* University hospital of Turin (approval number 256/2022 Protocol No. 35286 approved on 17 Jun 2022) for studies involving humans.

## Consent

Written informed consent for publication of images was obtained and stored according to local law.

## Conflicts of Interest

The authors declare no conflicts of interest.

## Peer Review

The peer review history for this article is available at https://www.webofscience.com/api/gateway/wos/peer‐review/10.1111/cge.14713.

## Supporting information


Table S1.

## Data Availability

The data that support the findings of this study are available on request from the corresponding author. The data are not publicly available due to privacy or ethical restrictions.
